# From Bioinformatic Modeling to Clinical Observation: Potential Implications of Ribosomal RNA Folding in *Blastocystis* sp. Isolates from Symptomatic and Asymptomatic Carriers

**DOI:** 10.3390/pathogens14101009

**Published:** 2025-10-07

**Authors:** Fernando Martínez-Hernández, Arony Martínez, Cecilia Zampedri, Mirza Romero-Valdovinos, Carlos Jiménez-Gutiérrez, Karina Flores-Martínez, Armando Trejo-Chávez, Guiehdani Villalobos, Pablo Maravilla

**Affiliations:** 1 Hospital General “Dr. Manuel Gea González”, Calzada de Tlalpan No. 4800, Mexico City 14080, Mexico; fherxyz@yahoo.com (F.M.-H.); arony@live.com.mx (A.M.); mirzagrv@yahoo.com (M.R.-V.); amcrr1962@gmail.com (C.J.-G.); 2 Department of Bioengineer, Escuela de Ingeniería y Ciencias, Instituto Tecnológico y de Estudios Superiores de Monterrey, Mexico City 14370, Mexico; mceciliazampedri@gmail.com; 3 Departamento de Patología, Facultad de Medicina Veterinaria y Zootecnia, Universidad Autónoma de Nuevo León, Ciudad General Escobedo 66054, Mexico; karinafloresm06@gmail.com (K.F.-M.); armandotrejoc@gmail.com (A.T.-C.); 4 Departamento de Producción Agrícola y Animal, Universidad Autónoma Metropolitana, Unidad Xochimilco, Mexico City 04960, Mexico

**Keywords:** *Blastocystis* sp., population structure, secondary structure of rRNA, Bud23, RPS5, RPS18

## Abstract

Here, 18S-rDNA sequences of *Blastocystis* sp., previously documented from symptomatic (cases) and asymptomatic (controls) carriers, were analyzed to determine their population structure, predict their secondary structure, and examine their interactions with ribosomal proteins (Bud23, RPS5, and RPS18). Phylogenetic and population differentiation analyses were performed using STRUCTURE software V2.3.4. Moreover, an analysis of the rRNA secondary structure and folding of each sequence was performed, and their probability of interaction with ribosomal proteins was determined. Phylogenetic and haplotype analyses sorted the sequences into genetic subtypes ST1, ST2, and ST3, while the population structure showed each cluster as a differentiated subpopulation, suggesting incipient speciation or cryptic species differentiation. Furthermore, the analysis of the secondary structure of rRNA exhibited specific arrangements for each subtype. In addition, the probability of interaction between 18S-rRNA sequences of *Blastocystis* from cases and controls with RPS5 and RPS18 was significant, matching the biological plausibility of the previously documented finding that control isolates had a lower generation time than isolates obtained from cases. These findings reinforce the hypothesis that ribosomal subtypes ST1–ST3 of *Blastocystis* represent evolutionarily distinct lineages with the potential to be recognized as future species. Furthermore, they underscore the functional relevance of 18S-rRNA sequences from clinical isolates of *Blastocystis*.

## 1. Introduction

*Blastocystis* sp. represents the most prevalent intestinal microeukaryote encountered in coproparasitological investigations worldwide. The prevalence of this organism exhibits significant geographic variation, with rates of 10–15% in developed nations compared to more than 50% in developing countries [1,2].

The pathogenicity of *Blastocystis* remains a subject of scientific debate. While numerous studies have established associations between *Blastocystis* colonization and various gastrointestinal and dermatological manifestations, other investigations suggest that it functions as a non-pathogenic commensal organism within the intestinal microbiota of asymptomatic individuals [3,4]. Furthermore, a remarkable characteristic of *Blastocystis* sp. is its extensive genetic diversity, with 44 distinct genetic subtypes (STs; ST1, ST2, ST3, … ST44) currently documented, all characterized through the analysis of small subunit ribosomal DNA (18S) sequences [5].

On the other hand, ribosomes serve as essential sites of protein synthesis across most organisms, functioning as complex particles primarily composed of ribosomal RNA (rRNA) and proteins. The eukaryotic ribosome consists of two distinct subunits (40S and 60S) that undergo an evolutionarily conserved assembly process. This process requires the precise arrangement of rRNA and more than 79 types of ribosomal proteins (RPs), coordinated by over 200 trans-acting biogenesis factors [6,7,8]. Within ribosomes, 18S-rRNA molecules form the structural core of the 40S subunit, folding into characteristic conformations, where small self-complementary regions create double helices and single-stranded hairpin structures. Research has demonstrated that specific regions of 18S-rRNA, through their secondary and tertiary configurations, facilitate the arrangement and assembly of various ribosomal proteins that constitute the ribosomal subunits [9]. Additionally, numerous investigations have utilized the secondary structures of ribosomal genes to validate or enhance phylogenetic reconstructions, highlighting their evolutionary significance [10,11]. Although 18S-rRNA is not commonly associated with pathogenicity, variation in its secondary structure can influence phenotype by tuning protein synthesis. Local shifts in 18S accessibility or conformation may alter prerRNA processing, small-subunit assembly, ribosomal protein recruitment, and translational efficiency, reshaping growth and environmental responses and, ultimately, traits linked to pathogenicity. Consistent with this view, temperature-dependent remodeling of the 18S structure and expression has been observed in *Plasmodium*, supporting a regulatory contribution of the rRNA structure to stage- and environment-specific phenotypes [12]. This mechanistic perspective leverages genomic evidence within an evolutionary framework, enabling phenotype-level explanations without relying on rigid virulence factor paradigms.

RPs are essential components that regulate protein synthesis and facilitate translation processes. However, numerous potential biological functions of these proteins remain unexplored [6]. Among the RPs most extensively investigated for their significant roles in diverse biological processes are Bud23, S5 (RPS5), and S18 (RPS18). It is important to note that Bud23, while not formally an RP, is a highly conserved methyltransferase that facilitates the critical processome-to-pre-40S transition during ribosome biogenesis [7,13].

The eukaryotic ribosomal protein RPS5 is located at the head of the 40S ribosomal subunit (5′ dominion) and belongs to a highly conserved ribosomal protein family. Beyond its fundamental role in translation, RPS5 exhibits significant extra-ribosomal functions. Research has demonstrated that RPS5 participates in cellular proliferation and apoptotic pathways [7,14] and contributes to hepatic pathophysiology [15].

Ribosomal protein RPS18 functions as a critical housekeeping component that specifically binds to 18S-rRNA, thereby stabilizing its structure and facilitating the assembly of the 40S subunit in eukaryotic cells. This protein plays an essential role in the binding of fMet-tRNA, which is crucial for translation initiation. Beyond its primary function in protein synthesis, RPS18 exhibits additional roles in various organisms, including modulation of bacterial growth patterns [16,17].

Previous research conducted on *Blastocystis* stool cultures compared isolates from patients experiencing gastrointestinal disorders (cases) with those from asymptomatic carriers (controls). This investigation revealed that isolates from symptomatic patients demonstrated significantly reduced growth rates and decreased nucleotide variability compared to those from asymptomatic individuals. Despite these physiological differences, Bayesian phylogenetic analysis successfully identified subtypes ST1, ST2, ST3, and ST7 across all isolates, but failed to establish distinct phylogenetic clusters that separated cases from controls [18].

The present study aims to conduct a comprehensive analysis of the population structure in conjunction with phylogenetic network assessment, while also predicting the secondary structure and folding patterns of 18S-rRNA in *Blastocystis* sp. isolates. Furthermore, we investigate potential interactions between these RNA structures and ribosomal proteins, utilizing previously documented sequence data obtained from both symptomatic and asymptomatic carriers to elucidate possible molecular mechanisms underlying pathogenicity.

## 2. Materials and Methods

### 2.1. Sequence Data

For this study, we obtained 18S-rDNA sequence data from 96 *Blastocystis* sp. isolates (49 from symptomatic patients and 47 from asymptomatic carriers) previously documented by Vargas-Sanchez et al. [18]. Sequences were retrieved from GenBank (https://www.ncbi.nlm.nih.gov/genbank/, accessed on 3 February 2025) using accession numbers KP055659–KP055754, along with corresponding clinical metadata. These sequences map to the 5′ region of the complete 18S-rDNA gene, approximately positions 26 to 340.

### 2.2. Sequence Alignments

We implemented SSU-ALIGN v0.1.1 [19] in DNA mode with model guidance to align study sequences against a *Blastocystis*-specific consensus model derived from curated full-length references across all subtypes and sequences from animals not included in the subtype system [20]. Secondary structure support was estimated with RNAalifold v2.4.17 [21] using non-default settings (aln, color, dangles = 2) across five temperatures (37–41 °C), and covariance models were built with ssu-build [19], enforcing an 80% gap threshold (gapthresh 0.8) while disabling relative-entropy filtering (enone). To reduce spurious positions, we compared three masking schemes in ssu-mask [19]—strict (pf 0.95, pt 0.95), medium (pf 0.90, pt 0.92; key “med90”), and relaxed (pf 0.85, pt 0.90)—and retained the medium mask for downstream analyses due to its balance of coverage and structural consistency.

Study sequences were then aligned against this model, producing structurally consistent alignments that account for conservation at the sequence and structural levels, providing the foundation for subsequent analyses.

### 2.3. Phylogeny and Haplotype Network Analysis

A median-joining network analysis was carried out using NETWORK 4.6 software [22], and haplotype networks were depicted under default settings and assumptions. For all analyses, the following sequences of *Blastocystis* available in GenBank were used as controls of subtypes: ST1: HQ641595-6; ST2: HQ641602, HQ641654, and JX305874-5; ST3: JX305879, HQ641613, JX305880, JX305883, and HQ641611.

To compare the match between phylogenetic *Blastocystis* subtypes and the population analysis, a Bayesian inference was performed using Mr. Bayes 3.1.2 program [23] for 10 million generations with sampling trees every 100 generations. Inferences that reached the stationary phase were collected and used to build a consensus tree.

### 2.4. Population Structure Analysis

The STRUCTURE analysis with software V2.3.4 [24] was carried out to determine the most probable number of clusters across all *Blastocystis* samples with each ST. The value of K, representing the theoretical number of independent populations (considering two groups—cases and controls—and three STs), was established using the predetermined values of the software: correlated allele frequencies and admixture [25].

The algorithm starts with an initial random association of alleles into K clusters and was executed with 10 independent replicates for each value of K from 1 to 10 using a burn-in period of 10,000 and 100,000 repetitions after burn-in. The appropriate number of clusters was determined by calculating the delta K value [25]. A second run was performed using the delta K value assigned to the program (K = 2 or K = 3). These new analyses were conducted with a burn-in period of 20,000 and 100,000 Markov chain Monte Carlo (MCMC) repetitions after burn-in [25].

### 2.5. RNA Secondary Structure and Folding

The 18S sequences of *Blastocystis* sp. from cases and controls (320 bp) were transcribed into 18S-rRNAs. Then, these fragments were inserted into consensus alignments of whole 18S-rRNA of *Blastocystis* (positions 22 to 352 in the 5′ domain, according to Spahn et al. [26] and Granneman et al. [27]) for STs 1–3. The secondary structures and folding were predicted under the RNAfold web server [28], and the minimum free energy (ΔG) was estimated for stability comparison.

### 2.6. Prediction of RNA–Protein Interactions

The interaction between 18S-rRNA sequences of *Blastocystis* from cases and controls and Bud23, RPS5, and RPS18 was analyzed; sequences of these RPs from ST1 and ST4 genomes of *Blastocystis* available in GenBank (S18: OAO11934 and XP 014528798.1; S5: OAO14796) were downloaded. Then, analysis of RNA–protein interactions was performed online using RPISeq software V1.0 [27], contrasting the converted sequences of each 18S-rRNA from cases and controls with the sequences of Bud23, RPS5, and RPS18 to predict the probability of interaction between them using a Support Vector Machine (SVM) algorithm.

### 2.7. Statistical Analysis

Student’s *t*-test was used to compare means for ribosomal proteins RPS5, RPS18, and Bud23, and Levene’s test was used to assess homogeneity of variance. Mean differences and their respective 95% confidence intervals (95% CI) were also analyzed. For the analysis of ST1, ST2, and ST3, proportions were tested using the X^2^ statistic, and compliance with the expected value assumption (e > 5) was verified. The analysis assumes a one-tailed hypothesis, with a significance level of *p* ≤ 0.05, with 95% CI. The analysis was performed using SPSS version 31 for iOS (SPSS, Chicago, IL, USA).

## 3. Results

### 3.1. Phylogenies and Population Structure Analysis

**Figure 1:**
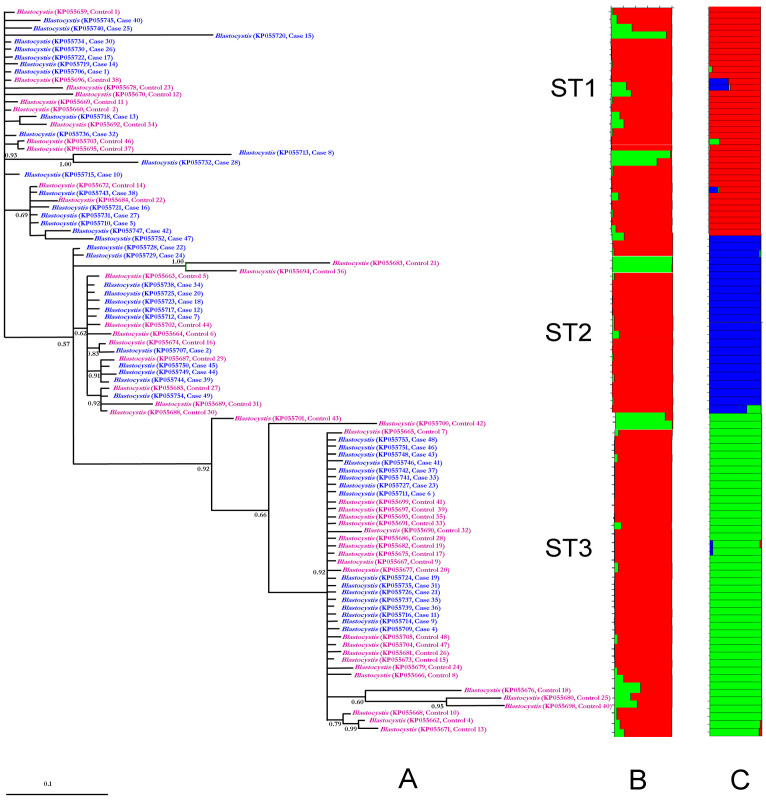
In (**A**), phylogenetic inference based on 18S-rDNA sequences of *Blastocystis* sp. [18] is shown. The numbers at the nodes indicate Bayesian posterior probabilities. Figure 1 illustrates the genetic structure clusters (denoted by different colors) derived from the 18S-rDNA sequences of *Blastocystis* sp. These clusters were generated through Bayesian Markov chain Monte Carlo (MCMC) simulation, considering either two mayor subpopulations (cases and controls) in panel B or three subpopulations (ST1, ST2, and ST3) in panel C; where the colors means a defined subpopulation and each sample is represented by a vertical bar segmented into colored portions, indicating membership probability in either K = 2 or K = 3 clusters. The probabilities of group membership for the case–control analysis (**B**) ranged from 0.86 to 0.99 for K1 and from 0.91 to 0.99 for K2, while under the assumption of three subpopulations (**C**), probabilities ranged from 0.58 to 0.99 for K1, from 0.91 to 0.99 for K2, and 0.73–0.99 for K3.

Phylogenetic analysis revealed distinct clustering patterns for ST1, ST2, and ST3, with no discernible segregation between symptomatic cases and asymptomatic controls (Figure 1A). Consistent with these findings, STRUCTURE analysis generated a histogram clearly delineating three genetically distinct subpopulations corresponding to each ST (Figure 1C). In contrast, when the analysis was conducted under the assumption of two subpopulations (cases versus controls), no significant clustering pattern emerged (Figure 1B).

**Figure 2:**
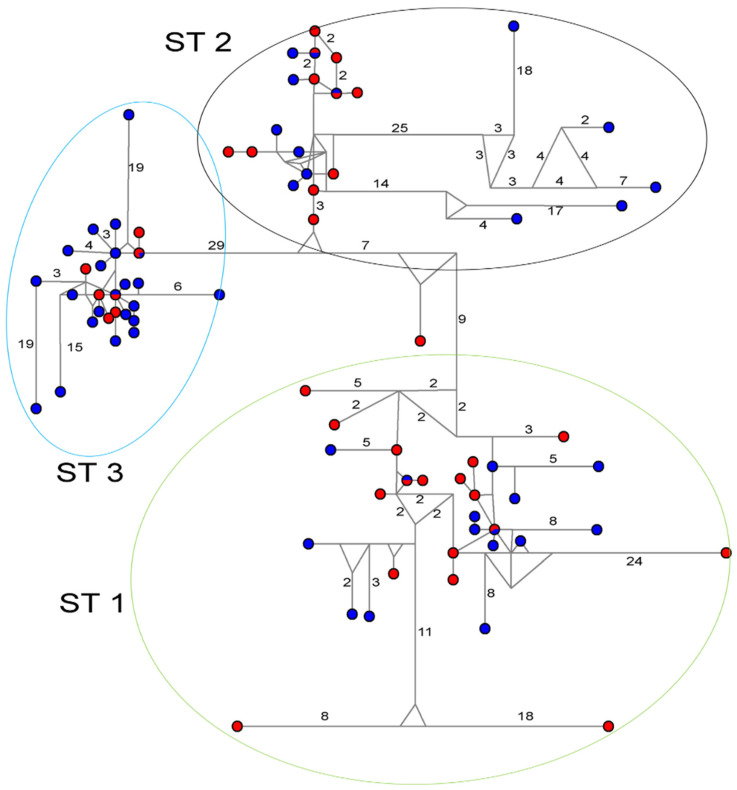
Haplotype network tree generated using 18S-rDNA sequences of *Blastocystis* sp. [18]. Sizes of circles are proportional to haplotype frequencies, and the numbers in branches are mutational changes. Blue indicates the control samples, and red indicates the case samples.

The haplotype network analysis corroborated the phylogenetic findings, with sequences clustering into specific groups corresponding to each of the STs (Figure 2). While this analysis similarly failed to differentiate between case and control haplotypes, it revealed identical haplotypes shared between symptomatic and asymptomatic carriers across all subtypes.

### 3.2. 18S-rRNA Secondary Structure and Conformational Analysis

**Figure 3:**
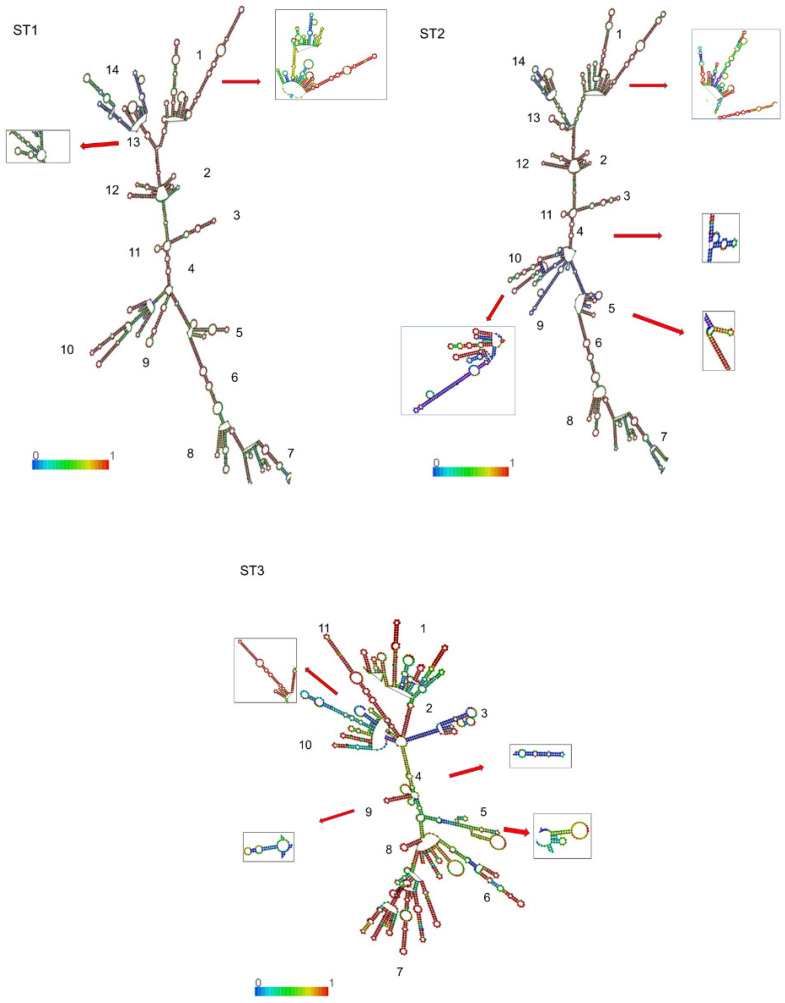
Inferred secondary structures of 18S-rRNA for ST1, ST2, and ST3; the structures are colored by base-pairing probabilities. The numbers at the bases of the branches show the ordering of the substructural loops. The colored line at the base represents the base-pair probability. Polymorphic conformational substructures are shown in boxes. The ΔG values of the thermodynamic ensemble ranged from −587.42 to −1192.10 Kcal/mol for ST1, from −541.50 to −577.10 Kcal/mol for ST2, and from −556.72 to −553.45 Kcal/mol for ST3.

As illustrated in Figure 3, the secondary structure of 18S-rRNA exhibited similar architectural frameworks across all STs, characterized by a conserved scaffold with subtype-specific variations throughout the molecule. Each subtype displayed a primary helix framework that was consistent between cases and controls, though with distinctive substructural elements and loops, i.e., small double strands or loop projections derived from the main structure. Following the nomenclature established by Van de Peer et al. [29] and Wuyts et al. [30], individual helices and substructural loops were enumerated in a clockwise orientation from the 5′ to the 3′ terminus. This analysis identified 14 distinct substructures in ST1 and ST2, while ST3 exhibited 11 substructures (Figure 3). Notably, ST1 displayed conformational polymorphism in substructures 1 and 13, whereas ST2 and ST3 each presented four conformational variants. Importantly, no case-specific or control-specific substructural elements were identified in any of the subtypes.

### 3.3. RNA–Protein Interaction Analysis

Based on the interaction probability data between ribosomal proteins (Bud23, RPS5, and RPS18) and 18S-rRNA sequences from *Blastocystis* isolates (Supplementary Materials, Table S1), we conducted comparative analyses between subtypes and between case and control groups. Table 1 summarizes these comparisons. All three ribosomal proteins demonstrated high interaction probabilities (mean > 0.91) with18S-rRNA, with Bud23 showing the highest values, followed by RPS18 and RPS5, respectively. The majority of the data points clustered between the lower quartile (Q1) and the median (Supplementary Materials, Figure S1). Notably, when comparing overall case versus control values, statistically significant differences were observed for RPS18 and RPS5 interactions; however, when stratified by subtype, no significant differences were detected between cases and controls within individual STs.

## 4. Discussion

*Blastocystis* STs are considered to be ribosomal linages [20], aligning with criteria established by Jacob et al. [31], which characterize ribosomal lineages as organisms with ≥80% of the 18S-rDNA gene sequenced, but lacking definitive morphological differentiation. These lineages may ultimately be reclassified as discrete, pending further morphological and molecular characterization. Our phylogenetic analyses and STRUCTURE histogram data provide robust support for the classification of ST1, ST2, and ST3 as distinct ribosomal lineages, offering compelling evidence for their status as cryptic species with genetically differentiated populations despite morphological homogeneity.

In human18S-rDNA, the copy number of ribosomal genes per cell demonstrates significant interpersonal variation, ranging from 67 to 412 copies (mean = 217) [32]. Comparatively, *Blastocystis* ST7 has been documented to contain 17 tandem copies [33], highlighting species-specific patterns of ribosomal gene organization.

The eukaryotic ribosomal RNA gene (rDNA) is structurally organized into multiple tandem-repeating units. These units of rDNA undergo coordinated evolution within species, resulting in homogenization of repetitive DNA sequences within a species while maintaining sequence divergence between species or higher taxonomic categories. This molecular evolutionary phenomenon is termed concerted evolution [34,35].

Concerted evolution provides a valuable framework for understanding cryptic speciation phenomena. For example, a phylogenetic investigation incorporating rRNA secondary structure analysis of eleven representative solefish specimens (family Soleidae) demonstrated that six species exhibited minimal variation, suggesting a concerted evolutionary pattern, while other samples suggested non-concerted evolutionary dynamics. Additionally, this analytical approach facilitated taxonomic clarification, resolving the previous misclassification of a genus [34].

In the present study, our analysis of the specific 18S-rRNA secondary structure arrangements for each subtype, coupled with the distinct subpopulation clustering observed in the STRUCTURE analysis, strongly suggests that concerted evolution drives the differentiation of *Blastocystis* subtypes. This evolutionary mechanism is consistent with the genomic organization of ribosomal genes in most eukaryotic organisms, where rDNA exists in tandem arrays and undergoes concerted evolution [34,36,37].

Our computational predictions of RNA–protein interactions revealed a hierarchical pattern of binding probabilities, with Bud23 demonstrating the highest interaction probability, followed by RPS18 and RPS5. Statistical analysis uncovered significant differences in RPS18 and RPS5 interaction probabilities between symptomatic and asymptomatic carriers in the overall sample. However, these differences were not observed when the interactions were stratified by subtype, suggesting subtype-specific interaction patterns. These findings align with the established functional roles of these ribosomal proteins. Bud23, as a highly conserved methyltransferase, functions during the early processome-to-pre-40S transition stage [7,13], making its interactions relatively resistant to 18S-rRNA sequence variations. Conversely, RPS18 and RPS5 directly bind to 18S-rRNA to stabilize its structure and facilitate 40S subunit assembly [16,17]. Consequently, sequence variations in 18S-rRNA more profoundly affect these later-stage interactions, potentially compromising assembly efficiency.

The bioenergetics of ribosome maturation represents a substantial cellular investment, with eukaryotic cells dedicating a majority of their metabolic energy to producing functional ribosomes [24]. The 5′ terminal region of 18S-rRNA exhibits considerable species-specific and cell type-specific variability [24]; nevertheless, its fundamental role in translation initiation and protein synthesis remains universally critical across eukaryotic lineages [6]. These functional constraints likely influence the evolutionary patterns observed in *Blastocystis* ribosomal RNA.

Previous research has documented that *Blastocystis* exhibits variable generation times (GT) in axenic culture, ranging from 8.5–19.4 h depending on strain, with a mean GT of 11.7 h across eight experimentally tested strains [38]. In an investigation conducted by Vargas-Sanchez et al. [18], fecal samples from symptomatic (cases) and asymptomatic (controls) *Blastocystis* carriers were cultured in two distinct media. This study revealed that isolates from symptomatic patients demonstrated significantly reduced growth rates compared to those from asymptomatic controls, suggesting enhanced protein synthesis efficiency in the control isolates. Furthermore, the controls exhibited greater nucleotide diversity relative to the case group. This observation aligns with established eukaryotic growth patterns, wherein hundreds of copies of transcriptional units are encoded during proliferative phases to accommodate increased ribosomal demand [39]. Consequently, greater sequence variation would be expected in samples displaying normal growth kinetics, such as those from the control group. These findings support the biological plausibility that subtle conformational alterations in ribosome assembly may substantially influence protein synthesis rates.

In our current investigation, the probability of interaction between RPS18 and RPS5 and 18S-rRNA was significantly higher in the control sequences compared to the case sequences. This molecular distinction may elucidate the observed reduction in generation time, manifesting as accelerated growth in control isolates relative to those from symptomatic cases. The enhanced RNA–protein interaction efficiency could potentially facilitate more rapid ribosomal assembly and consequently expedite protein synthesis in asymptomatic carriers.

It is noteworthy that ribosomal proteins may exhibit functions beyond their canonical roles in protein synthesis. For instance, a comprehensive functional characterization of RPS18 from the cattle tick *Rhipicephalus microplus* demonstrated that recombinant RPS18 significantly inhibited the growth of both Gram-negative and Gram-positive bacteria under in vitro conditions [16]. This finding suggests potential auxiliary functions of ribosomal proteins that may influence pathogen–host interactions in ways not directly related to translation efficiency.

While some studies have identified associations between symptomatic presentation and infection with specific *Blastocystis* subtypes [40,41], our findings do not support a direct correlation between virulence/pathogenicity and 18S-rRNA sequence composition. Although multiple studies have documented the release of factors that may induce inflammatory processes in the host [42,43,44], these pathogenic mechanisms have not been demonstrated to correlate with 18S-rRNA configuration or sequence variations.

Some limitations of the present study are as follows: First, complete 18S-rDNA sequencing was not performed, potentially underestimating the frequency of rRNA secondary structure folding polymorphisms. Second, pseudogene identification protocols were not implemented, introducing potential bias in the enumeration of substructure loops across STs. Third, there may be a potential sampling bias, because a small number of case and control samples were analyzed from a specific population.

## 5. Conclusions

Our findings reinforce the hypothesis that ribosomal subtypes ST1, ST2, and ST3 of *Blastocystis* represent evolutionarily distinct lineages with the potential to be recognized as future species. Furthermore, they underscore the functional relevance of 18S-rRNA sequences from clinical isolates of *Blastocystis*, suggesting the operation of concerted evolutionary processes in this organism. Furthermore, the differential interaction probabilities between *Blastocystis* 18S-rRNA secondary structures and ribosomal proteins RPS5 and RPS18 offer a mechanistic explanation for previously documented differences in generation times between isolates from symptomatic and asymptomatic carriers.

## Figures and Tables

**Table 1:** Probability of interaction determined by the Support Vector Machine (SVM) algorithm between RP sequences (S18, S5, and Bud23) and 18S-rRNA of *Blastocystis* for cases and controls.

	Cases (*n* = 46)	Controls (*n* = 45)	Mean Difference 95%IC	Level of Significance ^b^ Cases vs. Controls			
	Mean 95%IC ^a^ Median	Mean 95%IC Median		All Participants	ST1	ST2	ST3
RPS18 ^c^	0.940 0.935 to 0.945 0.940	0.947 0.942 to 953 0.960	−0.00734 −0.0146 to −0.0003	0.024	0.543	0.686	1
RPS5 ^d^	0.923 0.914 to 0.932 0.920	0.934 0.924 to 0.943 0.90	−0.01030 −0.0231 to 0.00251	0.057	0.386	0.713	0.467
BUD23 ^e^	0.984 0.983 to 0.986 0.990	0.986 0.985 to 0.988 0.990	−0.00211 −0.0044 to 0.00028	0.061	0.576	0.485	1

^a^ 95% Confidence intervals; ^b^ *t*-test, one-tailed hypothesis test with *p* ≤ 0.05; ^c^ Equality of variances is assumed: Levene’s test F = 0.207, *p* = 0.650; ^d^ Equality of variances is assumed: Levene’s test F = 0.125, *p* = 0.725; ^e^ Equality of variances is assumed: Levene’s test F = 2.569, *p* = 0.112.

## Data Availability

All relevant data are within the article.

## Supplementary Materials

The following supporting information can be downloaded at https://www.mdpi.com/article/10.3390/pathogens14101009/s1: Table S1: Values to predict the probability of interaction between the RPs (Bud23, RPS5, and RPS18) and the 18S-rRNA sequences of *Blastocystis* from each sample (case or control); Figure S1: Box plot.

## Abbreviations

The following abbreviations are used in this manuscript:

18S-rDNA	18S ribosomal DNA
18S-rRNA	18S ribosomal RNA
rRNA	Ribosomal RNA
ST	Subtype
RP	Ribosomal protein

## References

[1] Tan K.S. (2008). New insights on classification, identification, and clinical relevance of *Blastocystis* spp.. Clin. Microbiol. Rev..

[2] Kwon J.Y., Choi J.H., Lee H.I., Ju J.W., Lee M.R. (2024). Molecular Prevalence of *Blastocystis* sp. from Patients with Diarrhea in the Republic of Korea. Microorganisms.

[3] Olyaiee A., Sadeghi A., Yadegar A., Mirsamadi E.S., Mirjalali H. (2022). Gut Microbiota Shifting in Irritable Bowel Syndrome: The Mysterious Role of *Blastocystis* sp.. Front. Med..

[4] Stensvold C.R., Tan K.S.W., Clark C.G. (2020). Blastocystis. Trends Parasitol..

[5] Santin M., Figueiredo A., Molokin A., George N.S., Köster P.C., Dashti A., González-Barrio D., Carmena D., Maloney J.G. (2024). Division of *Blastocystis* ST10 into three new subtypes: ST42–ST44. J. Eukaryot. Microbiol..

[6] Qiu L., Chao W., Zhong S., Ren A.J. (2023). Eukaryotic Ribosomal Protein S5 of the 40S Subunit: Structure and Function. Int. J. Mol. Sci..

[7] Black J.J., Johnson A.W. (2022). Release of the ribosome biogenesis factor Bud23 from small subunit precursors in yeast. RNA.

[8] Rodgers M.L., Woodson S.A. (2021). A roadmap for rRNA folding and assembly during transcription. Trends Biochem. Sci..

[9] Ferreira-Cerca S., Pöll G., Gleizes P.E., Tschochner H., Milkereit P. (2005). Roles of eukaryotic ribosomal proteins in maturation and transport of pre-18S rRNA and ribosome function. Mol. Cell.

[10] Gillespie J.J., McKenna C.H., Yoder M.J., Gutell R.R., Johnston J.S., Kathirithamby J., Cognato A.I. (2005). Assessing the odd secondary structural properties of nuclear small subunit ribosomal RNA sequences (18S) of the twisted-wing parasites (Insecta: *Strepsiptera*). Insect Mol. Biol..

[11] Keller A., Förster F., Müller T., Dandekar T., Schultz J., Wolf M. (2010). Including RNA secondary structures improves accuracy and robustness in reconstruction of phylogenetic trees. Biol. Direct.

[12] Qi Y., Zhang Y., Mu Q., Zheng G., Zhang M., Chen B., Huang J., Ma C., Wang X. (2022). RNA Secondary Structurome Revealed Distinct Thermoregulation in *Plasmodium falciparum*. Front. Cell Dev. Biol..

[13] Black J.J., Sardana R., Elmir E.W., Johnson A.W. (2020). Bud23 promotes the final disassembly of the small subunit processome in Saccharomyces cerevisiae. PLoS Genet..

[14] Matragkou C.N., Papachristou E.T., Tezias S.S., Tsiftsoglou A.S., Choli-Papadopoulou T., Vizirianakis I.S. (2008). The potential role of ribosomal protein S5 on cell cycle arrest and initiation of murine erythroleukemia cell differentiation. J. Cell. Biochem..

[15] Xu W.H., Hu H.G., Tian Y., Wang S.Z., Li J., Li J.Z., Deng X., Qian H., Qiu L., Hu Z.L. (2014). Bioactive compound reveals a novel function for ribosomal protein S5 in hepatic stellate cell activation and hepatic fibrosis. Hepatology.

[16] Costa G.C.A., Silva F.A.A., Torquato R.J.S., Silva Vaz I., Parizi L.F., Tanaka A.S. (2024). Evaluation of the biological function of ribosomal protein S18 from cattle tick *Rhipicephalus microplus*. Ticks Tick-Borne Dis..

[17] Malygin A.A., Karpova G.G. (2010). Structural motifs of the bacterial ribosomal proteins S20, S18 and S16 that contact rRNA present in the eukaryotic ribosomal proteins S25, S26 and S27A, respectively. Nucleic Acids Res..

[18] Vargas-Sanchez G.B., Romero-Valdovinos M., Ramirez-Guerrero C., Vargas-Hernandez I., Ramirez-Miranda M.E., Martinez-Ocaña J., Valadez A., Ximenez C., Lopez-Escamilla E., Hernandez-Campos M.E. (2015). *Blastocystis* Isolates from Patients with Irritable Bowel Syndrome and from Asymptomatic Carriers Exhibit Similar Parasitological Loads, but Significantly Different Generation Times and Genetic Variability across Multiple Subtypes. PLoS ONE.

[19] Nawrocki E.P. (2009). Structural RNA Homology Search and Alignment Using Covariance Models. Ph.D. Thesis.

[20] Stensvold C.R., Clark C.G. (2020). Pre-empting Pandora’s Box: *Blastocystis* Subtypes Revisited. Trends Parasitol..

[21] Gruber A.R., Lorenz R., Bernhart S.H., Neuböck R., Hofacker I.L. (2008). The Vienna RNA website. Nucleic Acids Res..

[22] Bandelt H.J., Forster P., Röhl A. (1999). Median-joining networks for inferring intraspecific phylogenies. Mol. Biol. Evol..

[23] Ronquis F., Huelsenbeck J.P. (2003). MrBayes 3: Bayesian phylogenetic inference under mixed models. Bioinformatics.

[24] Pritchard J.K., Stephens M., Donnelly P. (2000). Inference of population structure using multilocus genotype data. Genetics.

[25] Evanno G., Regnaut S., Goudet J. (2005). Detecting the number of clusters of individuals using the software structure: A simulation study. Mol. Ecol..

[26] Spahn C.M., Beckmann R., Eswar N., Penczek P.A., Sali A., Blobel G., Frank J. (2001). Structure of the 80S ribosome from *Saccharomyces cerevisiae*—tRNA-ribosome and subunit-subunit interactions. Cell.

[27] Granneman S., Petfalski E., Swiatkowska A., Tollervey D. (2010). Cracking pre-40S ribosomal subunit structure by systematic analyses of RNA-protein cross-linking. EMBO J..

[28] Muppirala U.K., Honavar V.G., Dobbs D. (2011). Predicting RNA-Protein Interactions Using Only Sequence Information. BMC Bioinform..

[29] Van de Peer Y., De Rijk P., Wuyts J., Winkelmans T., De Wachter R. (2000). The European small subunit ribosomal RNA database. Nucleic Acids Res..

[30] Wuyts J., De Rijk P., Van de Peer Y., Winkelmans T., De Wachter R. (2001). The European Large Subunit Ribosomal RNA Database. Nucleic Acids Res..

[31] Jacob A.S., Busby E.J., Levy A.D., Komm N., Clark C.G. (2016). Expanding the *Entamoeba* Universe: New Hosts Yield Novel Ribosomal Lineages. J. Eukaryot. Microbiol..

[32] Smirnov E., Kalmárová M., Koberna K., Zemanová Z., Malínský J., Masata M., Cvacková Z., Michalová K., Raska I. (2006). NORs and their transcription competence during the cell cycle. Folia Biol..

[33] Poirier P., Meloni D., Nourrisson C., Wawrzyniak I., Viscogliosi E., Livrelli V., Delbac F. (2014). Molecular subtyping of *Blastocystis* spp. using a new rDNA marker from the mitochondria-like organelle genome. Parasitology.

[34] Liao D. (1999). Concerted evolution: Molecular mechanism and biological implications. Am. J. Hum. Genet..

[35] Gong L., Shi W., Yang M., Kong X. (2018). Characterization of 18S-ITS1-5.8S rDNA in eleven species in Soleidae: Implications for phylogenetic analysis. Hydrobiologia.

[36] Coen E., Strachan T., Dover G. (1982). Dynamics of concerted evolution of ribosomal DNA and histone gene families in the melanogaster species subgroup of Drosophila. J. Mol. Biol..

[37] Schlötterer C., Tautz D. (1994). Chromosomal homogeneity of *Drosophila ribosomal* DNA arrays suggests intrachromosomal exchanges drive concerted evolution. Curr. Biol..

[38] Zierdt C.H., Swan J.C. (1981). Generation time and growth rate of the human intestinal parasite *Blastocystis hominis*. J. Protozool..

[39] Eickbush T.H., Eickbush D.G. (2007). Finely orchestrated movements: Evolution of the ribosomal RNA genes. Genetics.

[40] Vogelberg C., Stensvold C.R., Monecke S., Ditzen A., Stopsack K., Heinrich-Gräfe U., Pöhlmann C. (2010). *Blastocystis* sp. subtype 2 detection during recurrence of gastrointestinal and urticarial symptoms. Parasitol. Int..

[41] Tan T.C., Suresh K.G., Smith H.V. (2008). Phenotypic and genotypic characterisation of *Blastocystis hominis* isolates implicates subtype 3 as a subtype with pathogenic potential. Parasitol. Res..

[42] Gonzalez-Arenas N.R., Villalobos G., Vargas-Sanchez G.B., Avalos-Galarza C.A., Marquez-Valdelamar L.M., Ramirez-Miranda M.E., Olivo-Diaz A., Romero-Valdovinos M., Martinez-Hernandez F., Maravilla P. (2018). Is the genetic variability of Cathepsin B important in the pathogenesis of *Blastocystis* spp.?. Parasitol. Res..

[43] Wawrzyniak I., Poirier P., Viscogliosi E., Dionigia M., Texier C., Delbac F., Alaoui H.E. (2013). *Blastocystis*, an unrecognized parasite: An overview of pathogenesis and diagnosis. Ther. Adv. Infect. Dis..

[44] Nourrisson C., Wawrzyniak I., Cian A., Livrelli V., Viscogliosi E., Delbac F., Poirier P. (2016). *Blastocystis* secreted cysteine proteases: A legumain-activated cathepsin B increases paracellular permeability of intestinal Caco-2 cell monolayers. Parasitology.

